# The relationship between personal and interpersonal mental health experiences and stigma-related outcomes in Hong Kong

**DOI:** 10.1192/bjo.2023.39

**Published:** 2023-04-17

**Authors:** Stephanie Ng, Hannah Reidy, Paul Wai-Ching Wong, Olga Zayts-Spence

**Affiliations:** School of English, The University of Hong Kong, Hong Kong; Department of Psychiatry, The University of Hong Kong, Hong Kong; and Mind HK, Hong Kong; Department of Social Work and Social Administration, The University of Hong Kong, Hong Kong

**Keywords:** Mental health, mental illness, stigma and discrimination, cultural sensitivity, Hong Kong

## Abstract

**Background:**

Previous research indicates that personal mental health experiences (e.g. one's current mental health status) and interpersonal mental health experiences (e.g. one's familiarity with someone with mental illness) are associated with stigma-related outcomes. These outcomes include knowledge, attitudes and desire for social distance from people with mental illness.

**Aims:**

To explore the extent to which current personal mental health status and familiarity with mental illness predict stigma-related outcomes in Hong Kong.

**Method:**

Data were drawn from a larger research project examining mental well-being in Hong Kong citizens. Citizens (*N =* 1010) aged ≥18 years were surveyed between August and September 2021.

**Results:**

Multiple regression analyses revealed that immediate family and friends showed better attitudinal outcomes and lower desire for social distance compared with people who did not know anyone with mental illness (all β > 1.00, all *P* < 0.05), whereas people with personal experience of mental illness showed higher prejudicial attitudes compared with people who did not know anyone with mental illness (β = −0.744, *P* = 0.016). Better current personal mental health predicted lower prejudicial attitudes (β = 0.488, *P* < 0.001) and mixed outcomes on different realms of mental health knowledge.

**Conclusions:**

Cultural concerns surrounding ‘saving face’ and emphasis on collectivistic values may explain the nonlinear relationship between personal and interpersonal mental health experiences and stigma-related outcomes. Future anti-stigma interventions should tailor their approaches to the needs of people with different levels of familiarity with mental illness and include efforts to support the mental health of the overall population.

Mental health stigma has been shown to have deleterious effects on the mental and physical health of people with mental health problems globally.^1–3^ The issue of stigma has been a topic of concern in the mental health field since the publication of Erving Goffman's seminal work, *Stigma: Notes on the Management of Spoiled Identity.*^4^ Despite such longstanding interest in this topic, there is a lack of consensus surrounding which mechanisms form and perpetuate stigma. On the one hand, stigma has been as an issue stemming from inaccurate or insufficient knowledge about mental health issues, or unfamiliarity with these issues leading to the labelling of people with mental health problems as ‘other’.^5^ On the other hand, there is evidence to suggest that mental health stigma is not merely a product of lacking knowledge or opportunities for contact, with studies suggesting that even people with high levels of knowledge and intimate relationships with people with mental illness can endorse high levels of stigma.^6^ The effects of these conceptual inconsistencies are evident in practical efforts to address stigma. Specifically, despite the implementation of numerous large-scale, anti-stigma efforts, these interventions have only been found to result in short-term behavioural and attitudinal change,^7^ and the ‘active ingredients’ (i.e. causal mechanisms leading to these changes) remain poorly understood.^8^

## The relationship between personal and interpersonal mental health experiences and stigma-related outcomes

Existing research demonstrates that mental health stigma is not merely a product of lacking knowledge or opportunities for contact. For instance, studies suggest that the relationship between familiarity and stigma-related outcomes is better represented as a U-shaped curve, as opposed to a linear relationship, whereby the highest levels of stigma are observed in people who do not know anyone with mental illness and people with the most intimate relationships with these individuals, such as immediate family members and mental healthcare providers.^6^ Furthermore, a recent review study of existing anti-stigma campaigns has presented the argument that stigma may resist change because individuals are motivated to sustain perceptions of individuals with mental illness as ‘other’.^5^ One way that these perceptions may be sustained is by drawing on subjective personal mental health experiences to justify these views, which allows the person to continuously revise their attitudes and perceptions based on situational factors.^9^

Taken together, these findings suggest that stigma is a complex and dynamic phenomenon that is meaningfully associated with factors such as personal and interpersonal experiences with mental health issues. Exploring the nuanced influences of these factors is, therefore, essential to enhance future theoretical and practical approaches to stigma.

## The present study

The present study explores the relationship between two variables related to personal and interpersonal mental health experiences (familiarity with mental illness and current personal mental health status) and stigma-related outcomes (knowledge, attitudes and desire for social distance) in the context of Hong Kong, using a representative survey sample of 1010 citizens administered in 2021. This investigation responds to a research direction that a growing number of scholars have begun to advocate for, which calls for a greater focus on ‘the context and variability of health-related behaviours’ as opposed to ‘programmatic or unified theories of change’.^5^ That is, it is essential for effective anti-stigma approaches to account for contextually specific factors that may influence how and why people have stigma.

Considerations surrounding one's influence and relative position within interpersonal relationships are particularly salient in Chinese-majority societies such as Hong Kong. Here, collectivistic values emphasise the preservation of one's interpersonal relationships, particularly with one's family members, which can have a significant impact on attitudes and behaviours toward people with mental illness.^10^ Specifically, people with family members with mental illness are likely to experience associative stigma, which refers to the phenomenon of someone being discriminated against because of their relationship with a stigmatised individual.^11^ Accordingly, we expect that immediate family members of people with mental illness in our survey sample will endorse the highest levels of stigma. Furthermore, research in Chinese contexts suggest that people with mental illness are likely to experience self-stigma because of the perceived burden on their family members.^10^ Thus, we expect that personal experience with mental illness will be correlated with higher attitudinal stigma, and that current mental health status and stigma-related outcomes will be inversely correlated in our sample; that is, people in better states of mental health will endorse lower stigma, and *vice versa*.

Although previous studies have investigated the topic of stigma in Chinese contexts,^10,12^ to our knowledge, no study has explicitly explored the relationship between personal and interpersonal mental health experiences and stigma-related outcomes within a large population sample. Furthermore, although a considerable number of past studies have operationalised stigma solely in terms of its attitudinal component,^13,14^ our study acknowledges the multifaceted nature of stigma by separately examining the influence of personal and interpersonal mental health experience variables on knowledge, attitudes and desire for social distance. We also retain the distinction between different familiarity categories during our analysis (i.e. self, immediate family, extended family, friend, colleague and do not know anyone) in contrast with previous studies that collapsed these categories (i.e. self, other, none) during analysis procedures.^15^

## Method

### Data source

The population survey used in this study was administered as part of a larger research project entitled ‘Mental Health in Hong Kong: Assessing Mental Well-Being, Mind HK Programs and Resources, and Mental Health Literacy, Support, and Stigma’ conducted in collaboration with a local mental health charity, Mind Mental Health Hong Kong Limited (Mind HK; charity number 91/16471). The project aims to examine measures of mental well-being in the Hong Kong population, to understand the factors that affect these measures (e.g. mental health stigma and resource availability) and to inform the development of improved mental health support systems and resources. The authors assert that all procedures contributing to this work comply with the ethical standards of the relevant national and institutional committees on human experimentation and with the Helsinki Declaration of 1975, as revised in 2008. This project has been approved by the Human Research Ethics Committee of The University of Hong Kong for the time period of 7 May 2020 to 31 December 2023 (reference number EA2006029).

### Respondents and data collection procedures

Survey data were collected using a random sampling method to avoid selection bias. In this approach, two lists of telephone numbers were generated. The first list included randomly selected numbers from telephone directories, and the second list was created based on the first list using the plus-minus one-two method. Duplicated numbers were removed, and all remaining numbers were ordered randomly in the final sample. Approximately 70% of numbers were mobile numbers and 30% were landline numbers.

Social Policy Research Limited, a Hong Kong-based research firm, was commissioned to conduct the surveys. The target population were Hong Kong citizens aged 18 or above who were able to speak Cantonese, Mandarin, or English. Interviews were conducted anonymously by interviewers who had undergone training to administer survey questions. Telephone calls were made on both weekdays and weekends. Informed consent was considered given following the respondent's verbal agreement to participate in the interview. When more than one eligible household member was available for the interview, the member whose birth month was the most proximal to the time of the call was interviewed. Interviews were considered successful when at least 90% of questions were answered. Each number was called five times before it was dropped as an unsuccessful case. Data quality was ensured using on-site monitoring and voice recording procedures.

The survey included 56 questions and took 20–30 min to complete with Web-CATI (Computer-Assisted Telephone Interview) technology. The branching of questions was guided by computer programs. On-site validation procedures, such as range and consistency checks, were utilised during the interviews. Data collection and validation procedures were conducted using SurveyCake, a cloud-based survey solution for enterprises (SurveyCake, Taipei, Taiwan; https://www.surveycake.com/en/why-surveycake/enterprise).

The survey was conducted between 23 August and 15 September 2021. The brief period of data collection was determined necessary because of the availability of funding. A total of 4000 numbers were sampled, with 1987 invalid numbers (i.e. fax/data line, non-residence line, non-working line) and 2013 valid numbers. Among the 2013 valid numbers, interviews were successfully completed for 1010 respondents, with a response rate of 50.2%. Using a 95% confidence level, the maximum sampling error of percentages based on the 1020 effective sample size would be less than ±3.1%.

Sample weighting of age and gender was applied to the data before the analysis, to correct for underrepresentation of sociodemographic groups. These weights were calculated based on 2021 mid-year census data (i.e. the data collected most proximally to the time of survey data collection), as reported by the Census and Statistics Department of The Government of the Hong Kong Special Administrative Region.^16^

### Measures

#### Measures of mental health knowledge, attitudes and behaviours

Three psychometrically validated scales measuring knowledge, attitudes and behaviours toward mental illness in community settings were included in the survey. All measurement scales have been empirically evaluated for reliability and validity for use in English.^17–19^ These measures are the same as the ones used in the ‘Time to Change’ survey in the UK, a collaborative project between an anti-stigma initiative (‘Time to Change’) and King's College London.^20^ The decision to use the same measures was taken to ensure that survey findings were commensurate with international research standards. To account for the dominant languages used in Hong Kong (English and Cantonese), items in these measures were translated and back-translated into Traditional Chinese by the research team members. All measures have been validated for use in Chinese-speaking populations.^21,22^

The first scale was the Mental Health Knowledge Schedule (MAKS), which is a 12-item measure assessing mental health knowledge that is separated into parts A and B.^17^ Part A assesses stigma-related mental health knowledge (e.g. appropriate forms of help-seeking for someone with a mental health problem) and part B asks respondents to classify whether various conditions are diagnosable mental illnesses or not. Responses are indicated on a five-point Likert scale from 1 (strongly disagree) to 5 (strongly agree). Total scores on the MAKS can range from 12 to 60, with higher scores indicating greater mental health literacy. The internal consistency of the MAKS is acceptable (*α* = 0.65). Scores on parts A and B were analysed separately.

The second scale was the Community Attitudes towards the Mentally Ill (CAMI), which is a 12-item measure examining attitudes toward mental illness, evaluating endorsements of statements surrounding sentiments of social exclusion, benevolence, tolerance and support for community mental healthcare.^18^ This scale can be separated into two subscales, namely the ‘Prejudice and Exclusion (CAMI-P/E)’ subscale and the ‘Tolerance and Support (CAMI-T/S)’ subscale. Responses are indicated on a five-point Likert scale from 1 (disagree strongly) to 5 (agree strongly). The highest possible total score on the CAMI is 60, with higher scores indicating more favourable attitudes toward individuals with mental illness. The internal consistency for both subscales are acceptable to good (*α* = 0.836 for the CAMI-P/E subscale, *α* = 0.729 for the CAMI-T/S subscale).^23^ Scores on the CAMI-P/E and CAMI-T/S subscales were analysed separately.

The third measure was the ‘Intended Behaviours’ subscale of the Reported and Intended Behaviour Scale (RIBS-IB), which consists of four questions assessing a respondent's willingness to engage with someone with mental illness in four key domains: living with, working with, living nearby and continuing a relationship with this person.^19^ The Intended Behaviours subscale has been used in past studies exploring the relationship between familiarity and desire for social distance.^15^ These questions are scored on a five-point Likert scale from 1 (strongly unwilling) to 5 (strongly willing), and consist of questions about intended future behaviours toward a person with mental illness (e.g. ‘In the future, I would be willing to live with someone with a mental health problem.’) The possible range of scores for the scale is between 1 and 6, with higher scores indicating higher willingness to approach someone with a mental illness. The internal consistency of the scale is good (*α* = 0.85).

#### Familiarity with mental illness

Respondents were asked to disclose their level of familiarity with mental illness by indicating their most proximal relationship with someone with a past or present mental illness (‘Who is the person closest to you who has or has had some kind of mental illness?’). Responses were coded numerically in descending order of familiarity: (1) immediate family, (2) partner (living together), (3) extended family, (4) partner (not living together), (5) friend, (6) colleague and (7) do not know anyone. For the purposes of analysis, the ‘partner (living together)’ category was collapsed with the ‘immediate family’ category, and the ‘partner (not living together)’ category was collapsed with the ‘extended family’ category. The decision to collapse these categories was made in alignment with the ‘level of contact’ report described by Holmes et al,^24^ which includes separate categories for people living with someone with mental illness, and people with a familial relationship with someone with mental illness but who do not live with that individual. Of note, people in the Hong Kong context usually live with their immediate family members because of high population density and limited housing space.^25^ For the purposes of analysis, we combined the ‘familiarity with mental illness’ variable with another question asking respondents to disclose whether they had ever been diagnosed with mental illness, and created a new variable with six familiarity categories, namely self, immediate family, extended family, friend, colleague and do not know anyone.

#### Current mental health status

Respondents were asked to disclose their current level of mental well-being (‘How would you describe your current mental health?’) Responses were scored on a five-point Likert scale from 1 (very poor) to 5 (very good). Current mental health status was treated as a numeric variable for analysis. Self-report measures of mental health have been used in past studies,^26^ and responses to these questions have been found to be associated with clinically significant symptoms and access to mental health treatment.^27^

#### Sociodemographic information

Demographic variables collected for the present study are gender, age, ethnicity and highest educational attainment. Gender was coded into a binary variable (female/male). Age was coded into six categories (18–19, 20–29, 30–39, 40–49, 50–59 and ≥60 years). Ethnicity was coded into two categories (Chinese or non-Chinese). Educational attainment was coded into seven categories (no schooling, primary, lower secondary, upper secondary, college diploma/certificate/associate degree, Bachelor's degree and Postgraduate). The ‘no schooling’ and ‘primary’ categories were collapsed into one category (‘primary and below’) for the purposes of analysis because of the small sample size of the ‘no schooling’ category (*n* = 6).

### Analysis

Descriptive statistics were first generated to explore the characteristics of the survey sample, reporting unweighted frequencies and weighted percentages for sociodemographic and personal mental health experience variables. Kolmogorov–Smirnov tests of normality were performed to determine whether subgroup scores for each outcome measure were normally distributed. These tests demonstrated that scores for most subgroups were not normally distributed (*P* < 0.001). Thus, the median was selected as the most appropriate measure of central tendency,^28^ and was reported along with s.d. and 95% confidence intervals for scores on each of the five stigma measures across subgroups of age, gender, educational attainment and the three personal mental health experience variables. These details were not reported for ethnicity subgroups because of the small sample size of non-Chinese respondents (*n* = 1).

Multiple regression analysis was used to examine the relationship between the two independent variables (familiarity with mental illness and current mental health status) and five stigma measures, controlling for age, gender and educational attainment. In block 1, age, gender and educational attainment were inputted as control variables. For the ‘familiarity with mental illness’ variable, dummy variables representing each of the subcategories were inputted in block 2, with the ‘do not know anyone’ category designated as the reference group. This category was selected as the reference group in line with previous literature that this group represents the lowest level of familiarity,^24^ and constitutes a baseline from which other familiarity groups can be compared against. ‘Current mental health status’ was treated as a numeric variable and inputted directly into block 2.

Statistical significance was evaluated using two-sided tests with a cut-off *P*-value of 0.05. Adjusted *R*^2^ was used as the measure of effect size, indicating the degree to which the overall regression model explained the amount of variance in stigma measure scores. Unstandardised β-coefficients were used to examine the strength of the effect of each predictor variable (i.e. subcategories of personal mental health variables) on stigma measure scores. Statistical analyses were performed with SPSS for Mac OS version 27.

## Results

Unweighted frequencies and weighted percentages of the sample across sociodemographic and personal mental health experience variables, as well as the median, s.d. and 95% confidence interval for each of the five stigma measures, are reported in Table 1. The majority of the sample identified as ethnically Chinese (99.8%, *n* = 1009). Most of the sample had completed upper secondary school or above (73.4%, *n* = 818). A vast majority of individuals indicated that they did not know anyone with a mental illness (70.1%, *n* = 701), and a notable portion of the sample reported personal experience with mental illness (15.4%, *n* = 157). Almost 80% of respondents reported that their current mental health status was ‘okay’ to ‘very good’ (79.2%, *n* = 816).

**Table 1:** Descriptive statistics for scores on stigma measures across sociodemographic and personal and interpersonal mental health experience variable subgroups

			RIBS-IB		CAMI-P/E		CAMI-T/S		MAKS part A		MAKS part B	
	*n* (unweighted)	% (weighted)	Median (s.d.)	95% CI	Median (s.d.)	95% CI	Median (s.d.)	95% CI	Median (s.d.)	95% CI	Median (s.d.)	95% CI
Gender	1010											
Male	452	44.7	13.00 (3.69)	12.39–13.07	18.00 (3.58)	17.91–18.58	22.00 (3.17)	22.05–22.64	23.00 (2.87)	22.55–23.08	22.00 (2.68)	21.62–22.12
Female	558	55.3	13.00 (3.90)	12.15–12.80	18.00 (3.60)	18.03–18.63	23.00 (3.38)	22.26–22.82	23.00 (2.71)	22.77–23.22	22.00 (2.64)	21.73–22.16
Age, years	1010											
18–19	29	1.6	13.29 (2.84)	11.83–14.78	18.74 (2.72)	17.30–20.14	23.00 (2.53)	21.43–24.07	22.00 (2.35)	20.91–23.36	22.00 (2.30)	20.64–23.03
20–29	150	12.7	14.00 (3.47)	12.95–14.16	19.00 (3.54)	19.13–20.37	23.00 (3.05)	22.35–23.42	23.00 (3.10)	22.04–23.12	22.00 (2.92)	21.71–22.73
30–39	240	17.6	13.00 (3.58)	12.19–13.25	19.00 (3.49)	18.35–19.38	23.00 (3.16)	22.25–23.18	23.00 (2.78)	22.82–23.65	23.00 (2.83)	21.72–22.56
40–49	212	17.9	14.00 (3.71)	12.79–13.88	19.00 (3.78)	18.29–19.40	23.00 (2.97)	22.41–23.28	24.00 (2.71)	23.09–23.88	22.00 (2.63)	21.83–22.61
50–59	196	18.4	13.00 (3.61)	11.95–12.99	18.00 (3.71)	17.47–18.54	23.00 (3.14)	22.09–23.00	24.00 (2.45)	23.07–23.79	22.00 (2.55)	21.12–21.86
≥60	183	31.8	12.00 (4.09)	11.74–12.19	17.00 (3.18)	16.88–17.58	22.00 (3.65)	21.45–22.25	23.00 (2.76)	21.99–22.60	22.00 (2.50)	21.46–22.01
Highest educational attainment	1010											
Primary and below	73	11.9	12.00 (4.02)	10.39–11.84	15.32 (3.37)	15.49–16.70	21.00 (3.41)	20.25–21.48	22.00 (2.78)	21.17–22.17	21.00 (2.29)	20.82–21.64
Lower secondary	119	14.7	12.00 (4.05)	11.20–12.51	18.00 (3.11)	17.38–18.39	22.00 (3.41)	21.54–22.64	23.00 (2.60)	22.18–23.03	21.00 (2.67)	20.90–21.77
Upper secondary	414	39.9	13.00 (3.81)	12.31–13.06	18.00 (3.49)	18.01–18.69	23.00 (3.25)	22.31–22.95	23.00 (2.84)	22.86–23.41	22.00 (2.61)	21.73–22.24
College	151	12.7	13.00 (3.62)	12.48–13.75	19.00 (3.48)	18.10–19.31	23.00 (3.25)	22.37–23.50	24.00 (2.70)	22.84–23.78	22.00 (2.74)	21.83–22.79
Bachelor's degree	236	19.3	13.00 (3.25)	12.89–13.81	19.00 (3.65)	18.80–19.83	23.00 (2.90)	22.45–23.27	23.00 (2.68)	22.81–23.56	23.00 (2.74)	21.85–22.63
Postgraduate	17	1.5	15.00 (3.36)	12.92–16.67	21.57 (3.47)	19.74–23.61	25.00 (2.87)	23.07–26.26	24.00 (2.28)	21.97–24.50	24.00 (2.72)	21.73–24.76
Ethnicity	1010											
Chinese	1009	99.8	N/A	N/A	N/A	N/A	N/A	N/A	N/A	N/A	N/A	N/A
Non-Chinese	1	0.2	N/A	N/A	N/A	N/A	N/A	N/A	N/A	N/A	N/A	N/A
Familiarity with mental illness	1010											
Self	157	15.4	14.00 (4.01)	12.11–13.38	18.00 (3.48)	17.14–18.25	22.00 (3.80)	21.64–22.85	23.00 (3.03)	22.18–23.14	21.00 (2.53)	21.23–22.03
Immediate family	45	4.5	14.00 (2.86)	13.23–14.95	19.00 (3.22)	18.54–20.48	23.00 (2.76)	22.44–24.10	24.00 (2.88)	22.45–24.18	23.00 (3.78)	20.53–22.79
Extended family	31	3.1	15.00 (2.79)	12.87–14.91	19.00 (3.31)	18.00–20.42	24.00 (2.65)	22.75–24.68	23.87 (2.51)	22.74–24.57	23.00 (2.51)	22.19–24.02
Friend	53	5.3	15.00 (3.38)	13.40–15.25	19.00 (4.06)	18.28–20.50	24.76 (2.76)	23.28–24.80	23.00 (2.08)	22.61–23.75	23.00 (2.28)	22.29–23.54
Colleague	16	1.6	14.69 (4.52)	9.98–14.75	19.94 (3.48)	17.03–20.71	21.84 (2.82)	20.74–23.72	23.21 (2.48)	22.32–24.94	24.00 (2.41)	22.60–25.14
Do not know anyone	708	70.1	12.00 (3.81)	12.00–12.56	18.00 (3.58)	17.95–18.47	22.00 (3.23)	22.04–22.51	23.00 (2.78)	22.67–23.08	22.00 (2.59)	21.62–22.01
Current mental health status	1010											
Very poor	56	5.6	13.00 (3.95)	11.14–13.24	17.00 (3.41)	16.60–18.42	22.74 (3.34)	21.32–23.10	23.00 (2.63)	22.22–23.62	23.00 (2.23)	22.09–23.28
Poor	138	15.2	12.00 (3.95)	11.12–12.38	17.00 (2.80)	16.43–17.32	22.00 (3.14)	21.46–22.47	22.00 (2.87)	21.85–22.77	22.00 (2.35)	21.79–22.54
Okay	269	26.4	13.00 (3.77)	12.32–13.23	18.00 (3.61)	17.92–18.79	23.00 (3.33)	22.34–23.15	23.00 (2.74)	22.45–23.11	22.00 (2.69)	21.45–22.10
Good	354	33.6	13.00 (3.56)	12.55–13.31	19.00 (3.66)	18.33–19.11	23.00 (3.25)	22.34–23.04	23.00 (2.60)	22.85–23.40	22.00 (2.59)	21.61–22.16
Very good	193	19.2	13.00 (4.04)	11.95–13.09	19.00 (3.75)	18.27–19.33	22.00 (3.34)	21.61–22.56	23.00 (3.06)	22.77–23.63	22.00 (3.01)	21.29–22.15

RIBS-IB, ‘Intended Behaviours’ subscale of the Reported and Intended Behaviour Scale; CAMI-P/E, ‘Prejudice and Exclusion’ subscale of the Community Attitudes towards the Mentally Ill; CAMI-T/S, ‘Tolerance and Support’ subscale of the Community Attitudes towards the Mentally Ill; MAKS, Mental Health Knowledge Schedule.

Table 2 shows the results of multiple regression analyses used to examine the relationship between each of the personal and interpersonal mental health experience variables and each of the five stigma measures, controlling for age, gender and educational attainment. Although all regression models were highly significant (*P* < 0.001), effect sizes for these models were small (*R^2^*_adjusted_ = 0.021–0.095). However, unstandardised β-coefficients indicated statistically significant relationships between the independent variables (familiarity with mental illness and current mental health status) and stigma-related outcome scores.

**Table 2:** Results of multiple regression analyses of the relationship between personal and interpersonal mental health experience variables and stigma outcome measures, controlling for age, gender and educational attainment

	RIBS-IB				CAMI-P/E				CAMI-T/S				MAKS part A				MAKS part B			
	*β* (unstandardised)	95% CI	Omnibus *F* (d.f.)	Adjusted *R*^2^	*β* (unstandardised)	95% CI	Omnibus *F* (d.f.)	Adjusted *R*^2^	*β* (unstandardised)	95% CI	Omnibus *F* (d.f.)	Adjusted *R*^2^	*β* (unstandardised)	95% CI	Omnibus *F* (d.f.)	Adjusted *R*^2^	*β* (unstandardised)	95% CI	Omnibus *F* (d.f.)	Adjusted *R*^2^
Familiarity with mental illness																				
Self	−0.079	−0.724 to 0.566			−0.744*	−1.346 to −0.142			−0.357	−0.919 to 0.205			−0.333	−0.814 to 0.148			−0.385		−0.844 to 0.074	
Immediate family	1.991***	1.061–2.920			1.110*	0.241–1.978			1.368**	0.557–2.179			0.465	−0.229 to 1.158			0.032		−0.630 to 0.693	
Extended family	0.664	−0.436 to 1.765			0.332	−0.696 to 1.360			0.527	−0.433 to 1.487			0.575	−0.246 to 1.396			0.628		−0.155 to 1.412	
Friend	1.790***	0.899–2.682			0.466	−0.367 to 1.299			1.048**	0.270–1.826			−0.128	−0.794 to 0.537			0.446		−0.189 to 1.081	
Colleague	−0.222	−1.851 to 1.406			0.029	−1.492 to 1.551			−0.504	−1.924 to 0.917			−0.356	−1.571 to 0.859			0.969		−0.190 to 2.129	
Do not know anyone	Reference/				Reference/				Reference/				Reference/				Reference/			
Covariates																				
Educational attainment	0.423***	0.193–0.653			0.516***	0.302–0.731			0.454***	0.253–0.654			0.385***	0.213–0.556			0.311***	0.148–0.475		
Age	−0.147	−0.346 to 0.051			−0.265**	−0.450 to −0.079			0.014	−0.159 to 0.187			0.104	−0.44 to 0.252			0.039	−0.102 to 0.180		
Gender	−0.275	−0.736 to 0.187	9.123 (8, 1001)***	0.061	0.116	−0.315 to 0.546	11.649 (8, 1001)***	0.078	0.210	−0.192 to 0.612	6.653 (8, 1009)***	0.043	0.243	−0.101 to 0.587	3.826 (8, 1001)***	0.022	0.119	−0.209 to 0.447	3.750 (8, 1001)**	0.021
Current mental health status	0.199	−0.005 to 0.404			0.488***	0.301–0.675			0.020	−0.158 to 0.197			0.205**	0.055–0.356			−0.170*	−0.314 to −0.026		
Covariates																				
Educational attainment	0.443***	0.211–0.674			0.531***	0.319–0.743			0.472***	0.270–0.673			0.397***	0.226–0.567			0.326***	0.163–0.489		
Age	−0.158	−0.358 to 0.042			−0.267**	−0.450 to −0.084			0.008	−0.166 to 0.182			0.110	−0.037 to 0.258			0.034	−0.107 to 0.174		
Gender	−0.224	−0.690 to 0.242	11.017 (4, 1005)***	0.038	0.089	−0.337 to 0.516	27.475 (4, 1005)***	0.095	0.258	−0.147 to 0.662	8.696 (4, 1005)***	0.030	0.220	−0.123 to 0.562	8.138 (4, 1005)***	0.028	0.151	−0.177 to 0.478	6.801 (4, 1005)***	0.022

*P-*values are two sided. RIBS-IB, ‘Intended Behaviours’ subscale of the Reported and Intended Behaviour Scale; CAMI-P/E, ‘Prejudice and Exclusion’ subscale of the Community Attitudes towards the Mentally Ill; CAMI-T/S, ‘Tolerance and Support’ subscale of the Community Attitudes towards the Mentally Ill; MAKS, Mental Health Knowledge Schedule.

*P < 0.05, **P < 0.01, ***P < 0.001.

Familiarity with mental illness significantly predicted variance in desire for social distance (RIBS-IB subscale), prejudice and exclusion attitudes (CAMI-P/E) and tolerance and support attitudes (CAMI-T/S), but not in levels of mental health knowledge (MAKS parts A and B). Individuals with immediate family members with mental illness showed lower desire for social distance (β = 1.991, *P* < 0.001), lower prejudice and exclusion attitudes (β = 1.110, *P* = 0.012) and higher tolerance and support attitudes (β = 1.368, *P* = 0.001) compared with the reference group (do not know anyone). Individuals with friends with mental illness showed lower desire for social distance (β = 1.790, *P* < 0.001) and higher tolerance and support attitudes compared with the reference group (β = 1.048, *P* = 0.008). People with personal experience of mental illness showed higher prejudice and exclusion attitudes compared with the reference group (β = −0.744, *P* = 0.016). The overall regression models testing the relationship between familiarity with mental illness and mental health knowledge (MAKS parts A and B) were significant, but the β-coefficients indicated that variance in scores was better explained by differences in educational attainment (*P* < 0.001).

Current mental health status significantly predicted variance in scores on prejudice and exclusion attitudes (CAMI-P/E) and mental health knowledge (MAKS parts A and B), but not on tolerance and support attitudes (CAMI-T/S) or desire for social distance (RIBS-IB). Higher levels of current mental health were associated with lower prejudice and exclusion attitudes (β = 0.488, *P* < 0.001), higher scores on the MAKS part A (β = 0.205, *P* = 0.008) and lower scores on the MAKS part B (β = −0.170, *P* = 0.021). The overall regression models testing the relationship between current mental health status and desire for social distance (RIBS-IB) and tolerance and support attitudes (CAMI-T/S) were significant, but the β-coefficients indicated that variance in scores was better explained by differences in educational attainment (*P* < 0.001).

## Discussion

The present study explored the relationship between personal and interpersonal mental health experiences and stigma-related outcomes in a large population sample, thus contributing to conceptual and practical approaches to mental health stigma. In contrast with results from the Attitudes to Mental Illness Survey conducted in the UK, which showed that higher familiarity is associated with improved stigma-related outcomes,^15^ our findings suggest a more nuanced relationship whereby immediate family members and friends of people with mental illness show lower levels of stigma, whereas people with personal experience of mental illness show significantly higher levels of stigma, compared with people who do not know anyone with mental illness. Our analysis of current mental health status and stigma-related outcomes suggest that better mental health status predicts lower prejudice and exclusion attitudes and mixed results on measures of mental health knowledge. Finally, although not the focus of our analysis, we found that educational attainment is a significant predictor of all stigma-related outcomes explored here. Overall, our findings indicate that the relationship between personal and interpersonal mental health experiences is not linear, and that future anti-stigma efforts would benefit from addressing the nuances of this relationship.

Contrary to our hypothesis that individuals with immediate family members with mental illness exhibit high levels of stigma because of factors such as associative stigma, our findings demonstrated that immediate family members have a lower desire for social distance, lower prejudice and exclusion attitudes and higher tolerance and support attitudes compared with people who do not know anyone with mental illness. Although it is possible that these findings corroborate previous findings that greater familiarity predicts lower stigma,^15^ another possible explanation is that these favourable responses reflect cultural values emphasising ‘saving face’ (i.e. preserving a respectable image in front of others).^29^ That is, these values may motivate immediate family members of people with mental illness to report more positive attitudes and behaviours toward people with mental illness, to protect the reputation of family members as well as their family name.

Our study also showed that intergroup differences in attitudes and desire for social distance are independent of levels of mental health knowledge, which were not significantly different between individuals from different familiarity categories. These findings are in line with past research on the relationship between contact and decreased prejudice, which indicates that increased empathy and reduced anxiety are more potent mediators of this relationship compared with increased knowledge about individuals in the outgroup.^30^ It is also aligned with the self-disclosure literature, which suggests that disclosing personal information and one's emotions (in contrast with merely sharing facts about oneself) are essential ingredients in developing relational intimacy and social connectedness.^31^ In the context of anti-stigma interventions, fostering a sense of familiarity and emotional intimacy can help target audience members to appreciate the ‘essential humanity of the individual with mental illness’.^6^

In support of our hypothesis, we found that personal experience with mental illness is associated with stronger attitudes of prejudice and exclusion. This is in line with previous research on self-stigma, which refers to the tendency for people with personal experiences of mental illness to internalise stigmatising attitudes and beliefs at the cost of their own well-being.^32^ Self-stigma may be prevalent in Hong Kong because of frequent experiences of mental health-related discrimination in interpersonal settings,^33^ combined with collectivistic values that increase the likelihood of one's internalisation of societal expectations and attitudes.^34^

In partial support of our hypothesis that current mental health status would be inversely correlated with stigma-related outcomes, our findings indicate that better personal mental health is associated with lower levels of prejudice and exclusion attitudes, and mixed results on subscales of mental health knowledge. In line with previous research that people with poor mental health may hold stronger attitudes of therapeutic pessimism,^15^ our findings suggest that better personal mental health is associated with a stronger belief that people with mental illness can recover and reintegrate into the community (as reflected in lower prejudice and exclusion attitudes). It is also likely that this relationship is bidirectional: people with greater confidence that recovery from mental illness is possible may be more likely to access mental health support resources themselves, thus reporting better states of current mental health.

Our findings regarding prejudice and exclusion attitudes can also explain our mixed findings surrounding the relationship between current mental health status and different forms of mental health knowledge. Specifically, the MAKS part A subscale assesses mental health knowledge related to the stigma of mental illness (e.g. whether people with mental illness can fully recover, whether most people with mental illness seek therapy), whereas the MAKS part B subscale assesses knowledge surrounding mental health diagnoses (i.e. whether people can accurately identify names of mental illness diagnostic categories). These findings suggest that although current mental health status is not associated with levels of factual knowledge surrounding mental illness, it is associated with a person's awareness of mental health stigma within the community.

Finally, our finding that educational attainment is a significant predictor of stigma-related outcomes is aligned with previous research.^35^ Previous findings have been equivocal, with some studies showing that lower education levels predict worse stigma-related outcomes,^36^ and others showing that stigma-related outcomes are worse among people with higher levels of education.^37^ Of note, previous research has discussed the role of cultural differences in explaining the differential influences of educational attainment on stigma-related outcomes,^35^ further underlining a need for future research to explore the relationship between culture and other correlates of stigma-related outcomes.

These findings suggest the need for anti-stigma interventions to use different approaches for populations with different levels of familiarity with mental illness.^6^ Interventions targeting the general public should aim to increase a sense of familiarity and emotional intimacy with people with mental illness, such as through facilitating positive and meaningful interactions with these individuals. Given the possibility that immediate family members of people with mental illness may be suppressing experiences of associative stigma or caregiver burden because of concerns surrounding 'saving face', it is also important for anti-stigma efforts to advocate for the development of supportive resources to address the affective and practical burdens affecting this population.^38^ Anti-stigma efforts targeted toward people with personal experiences of mental illness should support them in developing a sense of self beyond their diagnostic labels, such as by giving them opportunities to share their stories of lived experience in supportive contexts.^39^ Finally, given that current mental health status can have a notable influence on stigma-related outcomes, efforts to support the mental health of the general population should be considered an indispensable component of the anti-stigma agenda.

### Limitations and future directions

Several limitations of the present study can be noted. The ethnic distribution of our sample was biased toward ethnically Chinese respondents (99.8%), and is thus not representative of the overall Hong Kong population, which is composed of 91.6% ethnically Chinese individuals.^16^ Our study also only focused on two possible types of personal and interpersonal mental health experiences, which may explain the small adjusted *R*^2^ values of our regression models despite statistically significant β-coefficients. There were likely other explanatory factors at play that we were not able to explore in the present study.

Another limitation is that we did not conduct pairwise comparisons between familiarity groups, which may explain the insignificant results observed for certain groups (i.e. extended family and colleagues) in the regression model testing the relationship between familiarity with mental illness and stigma-related outcomes. That is, the differences between individual groups may have been overshadowed by larger effects within the overall regression model. Thus, future research should aim to conduct such pairwise comparisons (e.g. between family and friends, friends and colleagues, immediate and extended family members) so as to continue building a more nuanced depiction of the relationship between familiarity with mental illness and stigma-related outcomes. The cross-sectional design of the survey used in this study is also a limitation, as it does not allow for causal interpretations to be made from the current data. Finally, since self-report questions were used to inquire about respondents’ personal mental health experiences and mental health knowledge, attitudes and behavioural intentions, it is possible that social desirability and response biases affected the authenticity of responses. Future studies can use semi-structured interviews and focus groups to explore the rationales for participants’ responses via multiple-choice questions, and to explore findings that were contrary to our hypotheses (e.g. immediate family members showing lower levels of stigma).

Despite its limitations, our study meaningfully expands on existing theoretical and practical perspectives on mental health stigma. Moreover, although the details of study findings are specific to Hong Kong, the implications of these findings are applicable to global contexts. From a theoretical standpoint, we found that levels of mental health knowledge can operate independently from mental health attitudes and desire for social distance, and that factors such as personal and interpersonal mental health experiences and cultural norms can be associated with stigma-related outcomes. Future research should continue exploring how different factors operating at individual, interpersonal and societal levels affects different facets of stigma. From a practical standpoint, our findings suggest that first, it is crucial for anti-stigma interventions to foster emotional intimacy and a sense of familiarity in addition to providing factual information; second, that different approaches toward improving stigma-related outcomes should be used for people with different levels of familiarity with mental illness, and third, that anti-stigma interventions should be carried out in tandem with efforts to improve the mental health of the population. Of note, findings from this study will be incorporated into Mind HK's future programme direction, thus maximising the practical potential of the present research.

## Data Availability

The data that support the findings of this study are available on request from the corresponding author, O.Z.-S. The data are not publicly available due to their containing information that could compromise the privacy of research participants.
